# Effects of α‐synuclein pathology on synaptic dysfunction and clinical outcomes in normal aging

**DOI:** 10.1002/alz.71455

**Published:** 2026-05-03

**Authors:** Joseph R. Winer, Melanie J. Plastini, America Romero, Hillary Vossler, Isha Sai, Divya Channappa, Carla Abdelnour, Marian Shahid‐Besanti, Edward N. Wilson, Hamilton Se‐Hwee Oh, Christina B. Young, Alexandra Trelle, Maya Yutsis, Sharon J. Sha, Veronica Ramirez, Ryan Taylor, Kyan Younes, Tony Wyss‐Coray, Michael D. Greicius, Victor W. Henderson, Anthony D. Wagner, Kathleen L. Poston, Elizabeth C. Mormino

**Affiliations:** ^1^ Department of Neurology & Neurological Sciences Stanford University School of Medicine Palo Alto California USA; ^2^ Wu Tsai Neurosciences Institute Stanford University Stanford California USA; ^3^ The Phil and Penny Knight Initiative for Brain Resilience Stanford University Stanford California USA; ^4^ Nash Family Department of Neuroscience Icahn School of Medicine at Mount Sinai New York New York USA; ^5^ Brain and Body Research Center of the Friedman Brain Institute Icahn School of Medicine at Mount Sinai New York New York USA; ^6^ Department of Genetics and Genomic Sciences Icahn School of Medicine at Mount Sinai New York New York USA; ^7^ Ronald M. Loeb Center for Alzheimer's Disease Icahn School of Medicine at Mount Sinai New York New York USA; ^8^ Department of Radiology University of California Davis Sacramento California USA; ^9^ Department of Neurology University of California Davis Sacramento California USA; ^10^ Department of Epidemiology and Population Health Stanford University Stanford California USA; ^11^ Department of Psychology Stanford University Stanford California USA; ^12^ Molecular Imaging Program at Stanford (MIPS) Stanford University Stanford California USA

**Keywords:** co‐pathology, Lewy body, SAA, synaptic dysfunction

## Abstract

**INTRODUCTION:**

α‐Synuclein is the hallmark pathology of Parkinson's disease and dementia with Lewy bodies, described together as Lewy body disease (LBD). We investigated effects of α‐syn biomarker positivity in clinically unimpaired (CU) individuals.

**METHODS:**

We assessed α‐syn status (α‐syn ±) in 269 CU individuals using a cerebrospinal fluid (CSF) seed amplification assay (SAA). Fifty‐six participants with AD and 85 LBD spectrum participants were included for comparison. We compared α‐syn SAA results with demographics, fluid biomarkers, cognitive performance, and clinical measures.

**RESULTS:**

α ‐Syn positivity was detected in 9% of CU individuals, a lower rate than in clinically impaired participants with AD (16%) and LBD diagnoses (81%). Compared to α‐syn‐, α‐syn+ CU individuals were older, showed lower synaptic integrity, performed worse on tests of executive function and working memory, and reported more LBD‐related non‐motor symptoms.

**DISCUSSION:**

Further work is needed to understand the timeline of neural and clinical changes in α‐syn+ CU individuals and heterogeneity in disease progression.

## Highlights

- A total of 9% of clinically unimpaired older adults were α‐synuclein positive (α‐syn+) using cerebrospinal fluid (CSF) seed amplification assay (SAA).
- Synaptic dysfunction was elevated in α‐syn+ unimpaired older adults.
- The α‐syn+ status was associated with worse executive function test performance.
- The α‐syn+ unimpaired adults reported more non‐motor symptoms of Lewy body disease.
- Findings support the concept of preclinical Lewy body disease.

## 1 BACKGROUND

Parkinson's disease (PD) and dementia with Lewy bodies (DLB) are neurodegenerative syndromes collectively referred to as Lewy body diseases (LBD) that are pathologically characterized by the intraneuronal aggregation of misfolded α‐synuclein (α‐syn). In addition to motor and cognitive syndromes, individuals with α‐syn pathology can develop “prodromal” symptoms such as isolated rapid eye movement (REM) sleep behavior disorder and other non‐specific non‐motor symptoms.^1^ Critically, α‐syn pathology also occurs in the context of other neurodegenerative syndromes, such as Alzheimer's disease (AD).^2^ Given the heterogeneous clinical presentations seen in synucleinopathies, accurate biomarkers are crucial to understand the effects of underlying α‐syn pathophysiology across the clinical spectrum.^3^

Fortunately, recent developments allow for robust detection of α‐syn pathology in vivo through cerebrospinal fluid (CSF) α‐syn seed amplification assay (SAA)^4^ or skin biopsy.^5^ The α‐syn SAA is capable of detecting a single molecule of misfolded α‐syn, and shows high accuracy in detecting cortical Lewy body deposition in the context of LBD spectrum diagnoses compared to *post mortem* neuropathology.^6^, ^7^, ^8^ Such advances in highly‐specific biomarkers have motivated calls for biologically defining individuals found to have neuronal synuclein disease (NSD), anchored to markers of α‐syn aggregation and dopaminergic dysfunction, and staging according to clinical symptoms and functional impairment.^3^ Inherent to this proposed staging framework is the critical concept of a biomarker‐defined “preclinical” (or presymptomatic) stage of NSD. A precedent for this framework comes from the AD field, which conceptualizes clinically unimpaired (CU) individuals with biomarker evidence of abnormal β‐amyloid (Aβ) as a preclinical stage of AD.^9^ To date, studies leveraging CSF α‐syn SAA data suggest an α‐syn positivity prevalence between 8% and 16% in CU older individuals,^10^, ^11^, ^12^ which is similar to estimates from *post mortem* studies on the presence of Lewy bodies in the brain, regardless of location.^13^, ^14^, ^15^ Moreover, subtle effects related to cognitive and non‐cognitive LBD features have been explored in an emerging literature examining CU α‐syn+ individuals,^10^, ^11^, ^12^, ^16^ though reported effects and measures are not consistent across studies, highlighting the need for additional data characterizing the early effects of α‐syn positivity. α‐Syn SAA also presents the opportunity to characterize biomarkers of other neurodegenerative processes in the context of early α‐syn aggregation. For example, the recently characterized CSF YWHAG:NPTX2 ratio, a marker of synaptic integrity,^17^ has the potential to capture synuclein‐related synaptic and axonal deficits which are thought to precede neuronal loss in LBD.^18^, ^19^

We sought to expand our understanding of the early effects of α‐syn SAA positivity by leveraging our local cohort of CU older adults. We examined frequencies of α‐syn SAA positivity as well as associations with demographics, biomarkers of AD, synaptic dysfunction (specifically, CSF YWHAG:NPTX2), and neurodegeneration, and with cognitive test performance and clinical measures. We hypothesized that α‐syn+ CU older adults would (a) perform worse in cognitive domains typically associated with LBD, such as executive function, (b) have biomarker profiles indicating greater AD pathological burden and synaptic dysfunction, as well as (c) demonstrate non‐motor and motor symptoms related to LBD.

## 2 METHODS

### 2.1 Participants

We studied *N* = 415 participants from cohorts at Stanford University that collected CSF between 2010 and 2023, including the Stanford Alzheimer's Disease Research Center (ADRC),^20^ the Stanford Aging and Memory Study (SAMS),^21^ and the Pacific Udall Center (PUC).^22^ All participants underwent lumbar puncture and had α‐syn SAA data available. Data availability by modality is outlined for CU participants in Table S1.

Participants underwent diagnostic adjudication at multidisciplinary consensus meetings, which included a panel of neurologists, neuropsychologists, and research staff. CU participants were older adults (> 50 years old) who did not meet criteria for PD, mild cognitive impairment (MCI), or dementia based on history and neurological examination findings. Participants diagnosed clinically with MCI due to AD (AD‐MCI) or dementia due to AD (AD‐Dementia) met criteria for National Institutes of Health Alzheimer's Disease Diagnostic Guidelines^23^, ^24^ and were confirmed to have biomarker evidence of AD using CSF Aβ42:Aβ40 (see criteria below). PD was diagnosed using the UK Brain Bank criteria and required bradykinesia with muscle rigidity and/or rest tremor.^25^ Participants were further categorized as having PD with MCI (PD‐MCI) if they had a cognitive complaint and objective impairment on comprehensive cognitive testing using Movement Disorders Society (MDS) Level II criteria^26^ without substantial impact on functional activities. PD dementia (PDD) was defined as cognitive impairment severe enough to interfere with activities of daily living^27^ as determined by clinical history and the Clinical Dementia Rating.^28^ MCI due to Lewy bodies (MCI‐LB) and DLB were defined according to published criteria.^29^, ^30^ Study participants were grouped into three clinical etiological categories: CU, AD (AD‐MCI and AD‐Dementia), and LBD (PD without cognitive impairment, PD‐MCI, MCI‐LB, PDD, and DLB).

Five ADRC participants with α‐syn SAA results did not meet clinical criteria for CU, AD, or LBD. These participants were not included in analyses and are described in Table S2. The Stanford Institutional Review Board approved this study, and all study participants provided written informed consent.

**RESEARCH IN CONTEXT**

1. **Systematic review**: We conducted a review of the literature describing the effects of α‐synuclein (α‐syn) pathology in clinically unimpaired populations through PubMed until November 2025. We focused on studies which utilized cerebrospinal fluid α‐syn seed amplification assays (SAA).
2. **Interpretation**: α‐Syn SAA positivity was associated with several biomarker and clinical outcomes. The findings suggest that the presence of α‐syn pathology in clinically unimpaired older adults is associated with synaptic dysfunction, worse executive function, and non‐motor symptoms of Lewy body disease. We outline how these findings compare with other studies of α‐syn SAA in aging populations.
3. **Future directions**: Future analyses will follow the α‐syn+ individuals to determine rates of progression to Lewy body disease and worsening of symptoms. An enhanced understanding of the effects of α‐syn positivity will allow for the development of disease prevention strategies.

### 2.2 CSF collection and assessment of Aβ and tau

CSF collection procedures for ADRC, SAMS, and PUC followed a standard operating procedure so that all samples were collected, banked, and stored in a similar fashion. CSF was collected into polypropylene tubes by lumbar puncture before 11 AM. CSF was stored in 1.0 or 0.5 mL aliquots at −80°C until analysis. The fully automated Lumipulse G1200 instrument (Fujirebio US, Inc., Malvern, PA) was used to measure CSF p‐tau181, Aβ42, and Aβ40 as described previously.^22^, ^31^ In a dataset of 291 participants with a diagnosis of CU, AD‐MCI, or AD‐Dementia, a two‐cluster Gaussian mixture modeling approach was used to define a Aβ42:Aβ40 cutoff of 0.0943 (based on the 0.5 probability of belonging to the Aβ+ distribution).^31^ The p‐tau181 cutoff of 57.5 pg/mL was determined as two standard deviations above the mean of Aβ‐ CU in the current dataset. Aβ42:Aβ40 data were unavailable for 19 participants (16 CU, 3 LBD) and p‐tau181 data were unavailable for the same 19 as well as an additional two CU participants; these participants were excluded from all analyses that included AD biomarkers.

### 2.3 CSF α‐syn seed amplification assay

CSF samples were analyzed in Amprion Inc.’s CLIA‐certified (05D2209417) and CAP‐accredited (8168002) laboratory (San Diego, CA) in two batches that utilized the same methodology. See Supplementary Methods for an overview of the samples comprising the two batches. Clinical laboratory personnel who performed the α‐syn SAA were blinded to all clinical diagnoses and demographic data associated with the samples. Participants’ samples were analyzed using the qualitative version of α‐syn SAA that has been validated for clinical use under CAP/CLIA (SAAmplify‐αSYN) for the presence of misfolded α‐syn aggregates as previously described.^32^, ^33^ Briefly, each sample was run in triplicate using 40 µL CSF per well in a COSTAR 96 well plate with a final reaction volume of 200 µL. Each reaction mixture contained 0.3 mg/mL recombinant α‐syn in 100 mM PIPES pH 6.50, 500 mM NaCl, 10 mM ThT, and a 2.5 mm borosilicate glass bead per well. A baseline fluorescence reading was taken using a BMG LABTECH FLUOStar Ω Microplate Reader. Plates were then transferred to a TIMIX 5 shaker placed in an incubator set to 37°C and subjected to cycles of orbital shaking at 800 rpm for 1 min followed by 29 min of quiescent incubation for 7–10 days. Fluorescence readings were repeated once per day throughout the reaction period. Following final measurement, the maximum relative fluorescence units of each well was determined and the median of the three wells for each sample was calculated. CSF samples were classified as “detected” or “not detected” based on a preestablished threshold for the median maximum fluorescence of the triplicate wells. The highest raw fluorescence from each well was used in a probabilistic algorithm to establish whether each of the three replicates was a positive or negative readout. There were three possible outcomes: (1) if all three replicates from a sample were detected, the sample was classified as α‐syn+; (2) if zero or one of the replicates were detected, the sample was classified as α‐syn negative (α‐syn‐); (3) if two replicates were positive, the sample was deemed inconclusive. As a secondary classification criterion, samples with inconclusive results, defined by high replicate variability or low average maximum ThT fluorescence across the three replicates, were re‐evaluated and those falling below the established fluorescence threshold were classified as negative.

One CU sample was deemed inconclusive by Amprion, Inc. and was not included in analyses (see Supplementary Methods). Samples from two CU participants were classified by Amprion, Inc. as detected during an extended assay window beyond current validated specifications. We designated these two participants as α‐syn+ in all analyses.

### 2.4 CSF YWHAG:NPTX2 synaptic dysfunction measure

The SomaLogic (https://somalogic.com/) SomaScan v.4.0 assay was used to quantify the relative concentration of YWHAG (SeqId = 4179−57) and NPTX2 (SeqId = 6521−35) in CSF in samples obtained during the same lumbar puncture as the samples used for CSF SAA.^17^ Standard SomaLogic normalization, calibration, and quality control were performed on all samples, resulting in protein measurements in relative fluorescence units. The YWHAG:NPTX2 ratio was derived by log10‐normalizing the SomaScan protein levels then taking the difference between normalized YWHAG and NPTX2 values.^17^ SomaLogic data were available for 210 CU participants.

### 2.5 Plasma collection and assessment of GFAP and NfL

Ethylenediaminetetraacetic acid (EDTA) plasma was collected by venipuncture, centrifuged for 10 min at 2000×*g*, aliquoted in polypropylene tubes, and stored at −80°C until measurement. Samples were thawed on wet ice, centrifuged for 5 min at 4°C at 1000×*g*. Glial fibrillary acidic protein (GFAP) and neurofilament light (NfL) were measured on a Lumipulse G1200 instrument (Fujirebio US, Inc.). Plasma GFAP and NfL were available from 183 CU participants, with blood draw taking place within 1 year of the lumbar puncture used for CSF SAA.

### 2.6 Genetics data

Apolipoprotein E (*APOE*) genotyping was determined from whole‐genome sequencing (WGS) or obtained from National Cell Repository for Alzheimer's Disease (NCRAD) using a Fluidigm fingerprint panel. WGS was performed at the Beijing Genomics Institute in Shenzhen, China, or sequenced as part of the Stanford Extreme Phenotypes in AD project with sequencing performed at the Uniformed Services University of the Health Sciences (USUHS) on an Illumina HiSeq platform. The Genome Analysis Toolkit (GATK) workflow Germline short variant discovery was used to map genome sequencing data to the reference genome (GRCh38) and to produce high‐confidence variant calls using joint‐calling.^34^ *APOE* genotype (ε2/ε3/ε4) was determined using allelic combinations of single nucleotide variants rs7412 and rs429358, and was available for 339 participants (217 CU, 44 AD, and 78 LBD).

### 2.7 Cognitive and clinical assessments

Since neuropsychological batteries differed between study designs (ADRC, SAMS, PUC), analyses focused on cognitive tests common to CUs across all cohorts: Digit Span forward and backward (*N* = 240), Trail Making Test (Trails A duration, *N* = 262; and Trails B‐A, *N* = 261), Hopkins Verbal Learning Test—Revised (HVLT‐R) Delayed Recall (*N* = 240), and semantic fluency (*N* = 264). Digit Span data were comprised of Uniform Data Set Number Span in Stanford ADRC and PUC and WMS‐III Digit Span in SAMS, so WMS‐III scores were capped at the maximum Number Span scores of 14 for forward and 12 for backward.

Non‐motor and motor symptom severity were evaluated in a subset of participants using the MDS‐sponsored revision of the Unified Parkinson's Disease Rating Scale (MDS‐UPDRS). MDS‐UPDRS scores were available from *N* = 82 (*N* = 7 α‐syn+) CU participants for Part I (non‐motor experiences of daily living), *N* = 81 (*N* = 6 α‐syn+) CU for Part II (motor experiences of daily living), and *N* = 95 (*N* = 8 α‐syn+) CU for Part III (motor examination). Neuropsychiatric symptoms were evaluated in a subset of participants using the Neuropsychiatric Inventory‐Questionnaire (NPI‐Q; *N* = 194 [*N* = 19 α‐syn+] CU). Item severity scores were summed to create a total score representing overall neuropsychiatric symptom severity.

### 2.8 Statistical analysis

All statistical analyses were restricted to CU. We performed logistic regression analyses to determine associations between demographic variables, AD biomarkers, and α‐syn SAA status. Logistic regression was also used to determine odds ratios for α‐syn positivity predicting Aβ and tau positivity, and for *APOE*‐ε2 and ε4 dosage predicting α‐syn positivity, all adjusting for age and sex. Linear regression was used to examine associations between fluid biomarkers and α‐syn SAA status, with age and sex included as covariates. To investigate the relationship between α‐syn positivity and cognition, we performed linear regression analyses with cognitive test scores as the dependent variable and α‐syn status, age, sex, years of education, and CSF p‐tau181 as independent variables. For clinical assessments, MDS‐UPDRS scores and NPI‐Q total scores were dependent variables in linear regression models with α‐syn SAA status, age, and sex as independent variables. We considered two‐sided *p* < 0.05 to be statistically significant. No multiple comparisons correction was performed. All statistical analyses were performed in R version 4.4.1.

## 3 RESULTS

### 3.1 Participants and frequency of α‐syn pathology

Participant demographics for CU (*N* = 269) are summarized in Table 1 and demographics for AD (*N* = 56) and LBD (*N* = 85) groups are summarized in Tables S3 and S4. Frequency of α‐syn positivity is visualized together with the frequency of Aβ and tau positivity across all diagnostic groups in Figure S1. CSF α‐syn SAA determined that 24 (8.9%) CU, four (13.8%) AD‐MCI, and five (18.5%) AD‐Dementia participants were α‐syn+. Within the clinical LBD groups, 69 (81.1%) were α‐syn+; 87 (32%) CU and 35 (41%) LBD participants were Aβ+ by CSF Aβ42:Aβ40 ratio, and 42 (16%) CU, 20 (69%) AD‐MCI, 25 (93%) AD‐Dementia, and 18 (21%) of LBD participants were tau+ by CSF p‐tau181.

**TABLE 1:** Clinically unimpaired participant demographics.

α‐Synuclein SAA status	Negative	Positive
No. (%)	245 (91.1%)	24 (8.9%)
Age (years)	68.4 (6.77)	72.3 (6.82)
No. (%) female	146 (59.6%)	11 (45.8%)
Years of education	16.9 (2.18)	16.8 (2.50)
Missing	26 (10.6%)	1 (4.2%)
N (%) AD status		
A‐T‐	144 (58.8%)	11 (45.8%)
A+T‐	47 (19.2%)	7 (29.2%)
A‐T+	8 (3.3%)	1 (4.2%)
A+T+	29 (11.8%)	4 (16.7%)
Missing	17 (6.9%)	1 (4.2%)
N (%) APOE genotype		
ε2/ε3	22 (9.0%)	1 (4.2%)
ε2/ε4	4 (1.6%)	0 (0%)
ε3/ε3	121 (49.4%)	9 (37.5%)
ε3/ε4	44 (18.0%)	5 (20.8%)
ε4/ε4	7 (2.9%)	4 (16.7%)
Missing	47 (19.2%)	5 (20.8%)

*Note*: Participant characteristics based on clinical group and α‐synuclein status. Mean and standard deviation shown for age and years of education. Alzheimer's disease status assessed with CSF β‐amyloid 42:β‐amyloid 40 (cutoff < 0.0943) and p‐tau181 (cutoff > 57.5 pg/mL).

Abbreviations: AD, Alzheimer's disease; APOE, apolipoprotein E; A/T, β‐amyloid/tau; CSF, cerebrospinal fluid; SAA, seed amplification assay.

### 3.2 Associations with demographics, biomarkers, and *APOE* genotype

**FIGURE 1:**
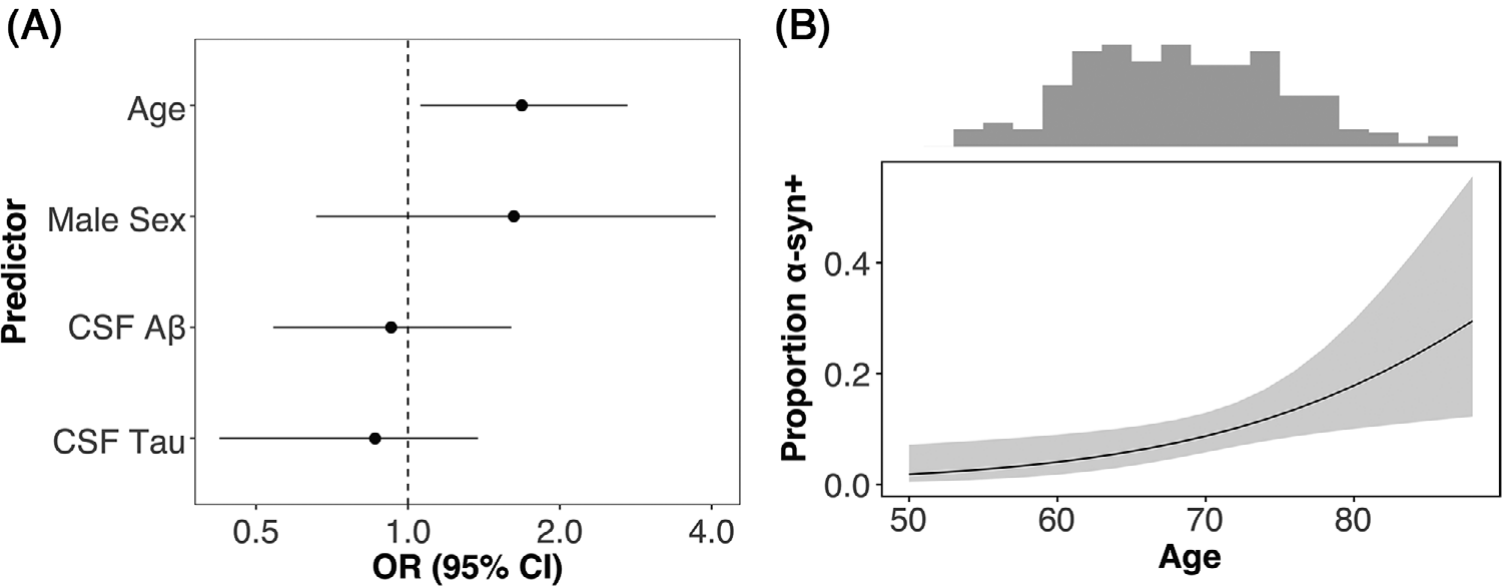
α‐Synuclein status associations with demographics, β‐amyloid, and tau in clinically unimpaired participants. (A) Forest plots show adjusted OR and 95% CI predicting α‐synuclein positivity for clinically unimpaired participants from a logistic regression model. (B) Proportion of α‐synuclein positivity with increasing age, with 95% CI. Histogram (top) illustrates density of participants across the age range. CI, confidence interval; CSF, cerebrospinal fluid; CSF Aβ, CSF β‐amyloid 42:β‐amyloid 40 ratio; OR, odds ration.

**FIGURE 2:**
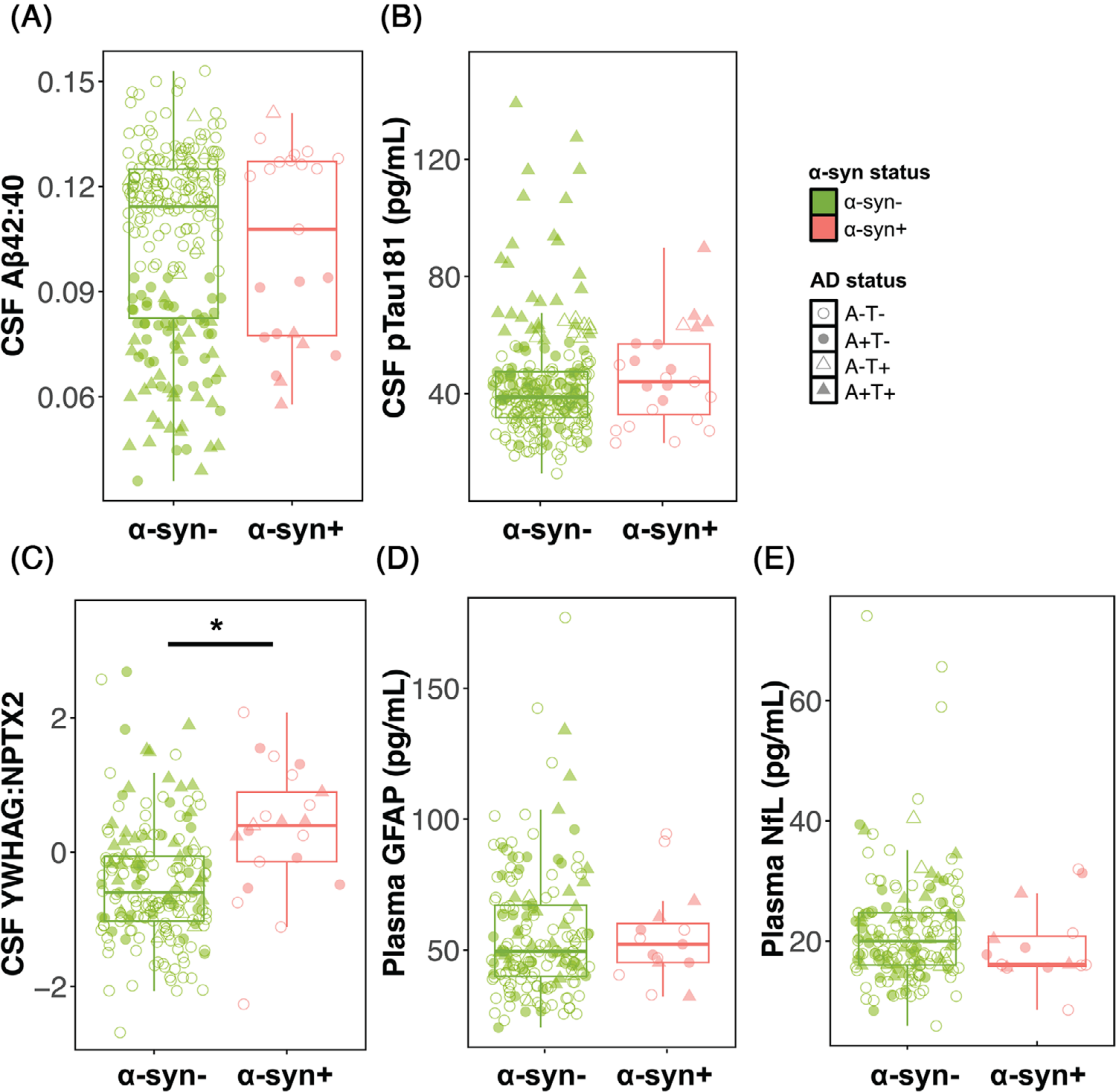
Associations between α‐synuclein status and continuous fluid biomarkers in clinically unimpaired participants. Boxplots show median and interquartile range in (A) CSF β‐amyloid 42:β‐amyloid 40 ratio, (B) CSF p‐tau181, (C) CSF YWHAG:NPTX2, (D) plasma glial fibrillary acidic protein, and (E) plasma neurofilament light for α‐synuclein positive and negative clinically unimpaired participants. Unadjusted data are displayed. Solid line indicates a significant difference in a linear model adjusting for age and sex. CSF p‐tau181 values for three α‐synuclein negative outliers (> 165 pg/mL) are omitted from the plot. CSF, cerebrospinal fluid; CSF Aβ, CSF β‐amyloid 42:β‐amyloid 40 ratio, GFAP, glial fibrillary acidic protein, NfL, neurofilament light.

All analyses were restricted to CU participants. First, we investigated associations with α‐syn SAA status using a logistic regression model that included age, sex, CSF Aβ42:Aβ40 ratio, and CSF p‐tau181 as predictors. In this model, only older age was a significant predictor of α‐syn SAA status (odds ratio [OR] 1.68, 95% confidence interval [CI] 1.06–2.72; Figure 1). Logistic regressions with dichotomous Aβ and tau status as predictors showed that neither Aβ positivity nor tau positivity were significantly associated with α‐syn SAA status (Aβ, OR = 1.60, 95% CI 0.65–3.89; tau, OR = 1.14, 95% CI 0.34–3.27). We additionally examined whether continuous CSF Aβ and tau differed by α‐syn SAA status. Linear regressions that included α‐syn SAA status, age, and sex as independent variables showed no significant association with α‐syn SAA status for either AD biomarker (Aβ42:Aβ40 ratio, unstandardized β ± standard error = 0.00 ± 0.01, *p* = 0.99; p‐tau181, −2.96 ± 5.55, *p* = 0.59; Figure 2A, B).

CSF YWHAG:NPTX2 was significantly elevated in α‐syn+ CU, representing an association between α‐syn aggregation and synaptic dysfunction (0.54 ± 0.18, *p* = 0.003; Figure 2C). In contrast, plasma GFAP and NfL did not differ by α‐syn SAA status (GFAP, −2.53 ± 6.14, *p* = 0.68, NfL, −2.55 ± 2.01, *p* = 0.21, Figure 2D, E).

Finally, *APOE*‐ε4 dosage was significantly associated with α‐syn positivity, and there was no association with *APOE*‐ε2 dosage (*APOE*‐ε4, OR = 2.56 95% CI 1.24–5.26; *APOE* ε2, OR = 0.46 95% CI 0.02–2.57). The effect of *APOE*‐ε4 dosage was attenuated but still significant when including Aβ status in the model (*APOE*‐ε4, OR = 2.35, 95% CI 1.02–5.54, Aβ status, OR = 1.19, 95% CI 0.36–3.77). Out of *N* = 21 *APOE*‐ε4/ε4 homozygotes in the full study sample, 10 (48%) were α‐syn+.

### 3.3 Associations with cognition

**FIGURE 3:**
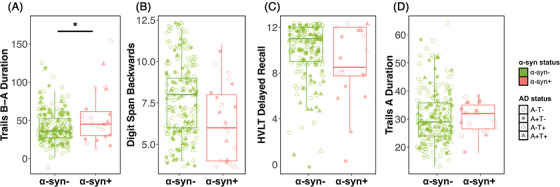
α‐Synuclein associations with cognitive test performance in clinically unimpaired participants. Boxplots show median and interquartile range in (A) Trail Making Test B‐A, (B) Digit Span Backwards, (C) HVLT‐R Delayed Recall, and (D) Trail Making Test A performance for α‐synuclein positive and negative clinically unimpaired participants. Unadjusted data are displayed. Solid line indicates significant difference in a linear model adjusting for age, sex, years of education, and CSF p‐tau181. CSF, cerebrospinal fluid; HVLT‐r, Hopkins Verbal Learning Test—Revised.

Test performance by α‐syn SAA status and linear regression results are displayed in Table S5. α‐syn positivity was associated with worse performance on Trails B‐A, a test of executive function (11.2 ± 4.96, *p* = 0.02; Figure 3A), and marginally negatively associated with Digit Span Backward, a test of working memory (−0.95 ± 0.52, *p* = 0.07; Figure 3B). p‐Tau181, but not α‐syn positivity, was associated with HVLT‐R Delayed Recall (−0.02 ± 0.01, *p* = 0.001; Figure 3C) and marginally associated with Trails A (0.05 ± 0.02, *p* = 0.07; Figure 3D). Semantic fluency and Digit Span Forward performance were not associated with either α‐syn SAA status or p‐tau181.

### 3.4 Associations with clinical outcomes

**FIGURE 4:**
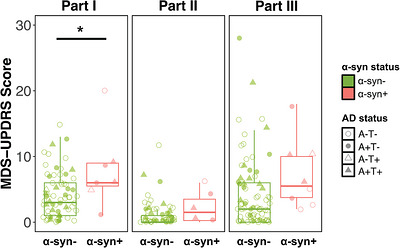
α‐Synuclein associations with Parkinson's disease symptom severity in clinically unimpaired participants. Boxplots show median and interquartile range in MDS‐UPDRS (A) Part I non‐motor symptoms, (B) Part II self‐reported motor symptoms, and (C) Part III motor exam for α‐synuclein positive and negative clinically unimpaired participants. Unadjusted data are displayed. Solid line indicates significant difference in a linear model adjusting for age and sex. One outlier is omitted from the Part III plot, a α‐synuclein negative participant with a score of 43. MDS‐UPDRS, Movement Disorders Society Unified Parkinson's Disease Rating Scale.

We next examined associations between CSF α‐syn status and LBD‐related clinical outcomes assessed using the MDS‐UPDRS and neurologist‐rated motor scores. In a subset of CU with MDS‐UPDRS data available, a linear regression revealed that α‐syn positivity was associated with higher severity in non‐motor symptoms, as assessed by MDS‐UPDRS Part I (3.80 ± 1.51, *p* = 0.01, Figure 4). Several MDS‐UPDRS Part I items differed on the basis of α‐syn status, with significantly higher severity of subjective cognitive impairment, hallucinations, apathy, and constipation associated with α‐syn positivity (*p*’s < 0.05, summarized in Table S6). In contrast to non‐motor symptoms, motor symptom scores (self‐rated MDS‐UPDRS Part II and neurologist‐rated MDS‐UPDRS Part III) were not significantly associated with α‐syn SAA status (Part II, 1.23 ± 0.80, *p* = 0.13; Part III, 2.00 ± 2.56, *p* = 0.44).

Finally, in a subset of CU who had neuropsychiatric symptom severity assessed with the NPI‐Q, α‐syn positivity was not associated with NPI‐Q severity (0.09 ± 0.28, *p* = 0.77).

## 4 DISCUSSION

In our cohort, 9% of CU, 14% of AD‐MCI, and 19% of AD‐Dementia participants were α‐syn SAA positive, versus 81% of individuals with LBD spectrum clinical diagnoses. We found that α‐syn+ CU adults were older, had elevated synaptic dysfunction (indicated by increased CSF YWHAG:NPTX2), and were more likely to carry an *APOE‐*ε4 allele relative to α‐syn‐ CU adults. Furthermore, α‐syn+ CU tended to perform worse on tests of executive function and working memory and reported more LBD‐related non‐motor symptoms. In contrast, α‐syn status in CU older adults was not significantly associated with CSF Aβ or tau, plasma GFAP or NfL, episodic memory performance, motor symptoms, or neuropsychiatric symptoms. These cross‐sectional findings, particularly the unique contributions establishing elevated synaptic dysfunction and an increase in UPDRS‐assessed non‐motor symptoms of LBD, represent an important addition to the growing literature characterizing the frequency and effects of α‐syn positivity in CU older adults assessed with CSF α‐syn SAA.

The frequency of α‐syn positivity in individuals above age 50 (the minimum age in our CU cohort) is estimated to be 9%−14% based on *post mortem* neuropathology studies, which includes those with brainstem only LBD pathology and not necessarily cortical pathology.^13^, ^14^ Given this low prevalence, large samples are necessary to identify sufficient asymptomatic α‐syn+ CU individuals to detect subtle effects of initial pathology. Furthermore, detection of in vivo α‐syn through SAA is currently only possible with CSF (in clinical practice), which is only available in select aging cohorts. Nine percent of the 269 CU participants in our study (mean age 69) were α‐syn SAA positive, a frequency similar to other published cohorts. To date, the BioFINDER study is the largest of α‐syn SAA in CU older adults, which found a frequency of 8% α‐syn+ in *N* = 1,182 older adults with a mean age of 70.^10^ Beyond BioFINDER, 16% of *N* = 576 CU (mean age 74) in the Alzheimer's Disease Neuroimaging Initiative (ADNI) were α‐syn+,^11^ and 9% of *N* = 378 CU (mean age 65) were α‐syn+ in aging cohorts from University of Wisconsin^12^ (also see smaller studies).^35^, ^36^, ^37^ The ADNI CU cohort was older and had higher frequency of Aβ positivity relative to the present Stanford cohort, BioFINDER, and the Wisconsin cohort, though it is not clear whether these factors might explain why the rate of α‐syn positivity is twice as high in ADNI. Regardless of differences across cohorts, it is notable that the 8%−16% CU α‐syn positivity frequency assessed with CSF α‐syn SAA in these four cohorts is similar to the 9%–14% frequency reported in the *post mortem* literature.^13^, ^14^ Collectively, the findings in our cohort and the previously published data from other cohort's support inclusion of α‐syn SAA positive individuals, who do not yet manifest neurological symptoms, within an integrated staging system for NSD (NSD‐ISS, stage 1).^3^ This prodromal group represents an opportunity to understand the effects of initial pathological processes relevant for LBD and future prevention strategies.

We found increasing frequency of α‐syn+ with increasing age in CU, replicating previous CSF α‐syn SAA findings.^10^ ^−^ ^12^ In our study, males were not significantly more likely to be α‐syn+ (OR 1.42, 95% CI 0.59−3.44, adjusted for age). In participants with AD, α‐syn+ rates were 14% in MCI and 19% in dementia in our cohort, compared to 19% in MCI and 28% in dementia in BioFINDER^38^ and 19% in MCI and 38% in dementia in ADNI.^11^ These differences may be due to participants at Stanford being more thoroughly evaluated for signs and symptoms indicative of clinical LBD (as this is a focus of the Stanford ADRC).^39^

Our study represents the first report that CSF YWHAG:NPTX2, a marker of synaptic integrity,^17^ is elevated in α‐syn+ CU individuals. This protein ratio, capturing upregulation of the neuronal protein YWHAG and downregulation of the synaptic homeostatic scaling factor NPTX2, was recently characterized as a biomarker of cognitive decline that is distinct from typical markers of AD and neurodegeneration.^17^ The observation that a measure of synaptic dysfunction is increased in cognitively healthy α‐syn+ individuals is in agreement with a literature suggesting that synaptic and axonal deficits precede neuronal loss in LBD.^18^, ^19^ In contrast, α‐syn positivity was not associated with differences in GFAP, a marker of astrocyte activation relevant in AD progression, or NfL, a marker of axonectomy and neurodegeneration. Together, this observed biomarker profile suggests that in CU older adults, α‐syn may aggregate in parallel or proximity with changes at the synapse^40^, ^41^ independent of AD progression or neurodegeneration. The causal timeline of these events is unknown, and future studies should leverage longitudinal measurement of YWHAG:NPTX2 and other synapse markers to determine whether synaptic changes precede and facilitate α‐syn aggregation, or whether α‐syn aggregation is the initial pathological change.

*Post mortem* studies indicate that α‐syn and AD pathology frequently co‐occur, both in individuals with clinical diagnoses of AD^42^, ^43^ and LBD,^44^, ^45^ and rodent studies suggest that Aβ may promote the spread of α‐syn along with tau.^46^ We observed that a greater percentage of Aβ+ CU were α‐syn+ relative to Aβ− CU, though this difference was not statistically significant. However, our α‐syn+ odds ratio of 1.81 in Aβ+ versus Aβ− CU is comparable to the effect observed in the larger BioFINDER (OR 1.72, adjusting for age) and ADNI studies (OR 2.03).^10^, ^11^ The effect for tau positivity predicting α‐syn SAA status also was not significant (OR 1.40). Tau positivity was not associated with α‐syn+ in CU in BioFINDER^10^ or in ADNI,^11^ although the Wisconsin study observed an association (30 of the 411 participants in that analysis had MCI, which may have influenced the association).^12^ Collectively, these findings support the efforts to include biomarkers of additional pathologies when considering a biological definition of neurodegenerative diseases, as now included in the revised criteria for diagnosis and staging of AD^9^ and highlighted as an outstanding research question that warranted further investigation in the NSD‐ISS.^3^

*APOE*‐ε4 is the strongest genetic risk factor for sporadic AD. *Post mortem* studies suggest that *APOE*‐ε4 carriers with LBD are more likely to have AD co‐pathology^47^ but not more likely to have LBD in isolation.^48^ We found that *APOE*‐ε4 dosage was associated with a higher likelihood of α‐syn positivity when adjusting for age, sex, and *APOE*‐ε2 dose, an effect that remained significant when adjusting for Aβ status. 48% of the 21 APOE‐ε4/ε4 homozygotes in our study were α‐syn+. Future studies should investigate the interactions between *APOE*, α‐syn, and AD across aging and cognitive decline.

Interestingly, we found that α‐syn+ CU performed worse than α‐syn− CU on tests of executive function and working memory even after adjusting for CSF p‐tau181. In contrast, p‐tau181 was associated with worse episodic memory performance. In contrast to memory changes typically observed in AD, executive function and visuospatial domains are more likely to be affected in clinical LBD.^26^, ^49^ In the BioFINDER study, α‐syn+ CU had worse cross‐sectional performance in global cognition as well as episodic memory performance and showed greater longitudinal decline in these domains in addition to attention/executive function.^10^ These patterns were similar when comparing individuals with and without AD pathology, suggesting a contribution of Lewy body pathology independent of AD. In ADNI, there were no cross‐sectional or longitudinal differences in CU cognitive performance by α‐syn SAA status.^11^ More work is needed to understand which cognitive domains are most sensitive to α‐syn pathology in the presymptomatic stage.

CU participants completed the MDS‐UPDRS, allowing for a unique comparison of non‐motor and motor symptoms on the basis of α‐syn SAA status. α‐syn+ CU participants endorsed significantly higher non‐motor symptom severity on the MDS‐UPDRS Part I relative to α‐syn− CU, suggesting that α‐syn positivity increases the likelihood of non‐motor symptoms in the preclinical phase. Self‐reported severity of cognitive impairment, hallucinations, apathy, and constipation were all significantly elevated in α‐syn+ CU participants, suggesting that these symptoms may serve as screening questions in the early detection of LBD. We did not observe a significant difference by α‐syn SAA status in self‐reported or neurologist‐assessed motor function (MDS‐UPDRS parts II & III) in CU, replicating findings in BioFINDER.^10^

One consideration in interpreting these findings is the sensitivity of CSF α‐syn SAA, which has been validated against *post mortem* results in a limited number of studies.^6^, ^7^, ^8^, ^11^, ^50^ *Post mortem* comparisons consistently show that α‐syn SAA has high sensitivity (∼100%) in detecting cortical Lewy bodies but only around 50% sensitivity in detecting brainstem and amygdala‐only LBD pathology (with the exception of individuals with REM‐sleep behavior disorder).^51^ The implication for examining α‐syn SAA+ CU individuals is that we may be missing as many as 50% of individuals in the early (pre‐cortical) pathological stages of LBD and therefore underestimating the prevalence of α‐syn positivity, and thus NSD‐ISS stage 1, in the broader population. Future availability of positron emission tomography (PET) ligands that bind to Lewy bodies will be critical for understanding the spatial distribution and spread of this pathology in CU.

An important limitation of our study was that it was cross‐sectional, and we did not have follow up data to determine whether individuals developed clinically specific LBD symptoms or declined over time. Previous studies indicate that α‐syn+ CU are likely to progress to PD or DLB^10^ and that α‐syn+ AD are more likely to develop LBD signs and symptoms.^52^ While existing cohorts with CSF α‐syn SAA data tend to focus either on the AD spectrum or LBD spectrum, future work could aim to bridge clinical categories to understand the effects of α‐syn positivity agnostic to current clinical diagnostic frameworks. Our study used a Clinical Laboratory Improvement Amendments (CLIA) certified, 7–10 day incubation version of the Amprion SAA protocol,^4^, ^33^ which may differ from protocols utilizing shorter incubation windows, although a recent comparison indicated no significant bias across protocols.^53^ One important limiting factor in interpreting our findings is that we did not correct for multiple comparisons in anticipation of subtle effect sizes in CU individuals, though our analyses focused on an a priori set of outcomes based on the LBD literature. Finally, the relatively small sample of α‐syn+ CU participants, particularly in the MDS‐UPDRS analyses, limits our statistical power and increases the risk of Type I and Type II errors. Future studies should confirm these associations in larger cohorts of CU participants. In identifying demographic, biomarker, cognitive, and clinical outcomes associated with α‐syn positivity in unimpaired older adults, our findings add converging evidence for including individuals with the earliest signs of α‐syn deposition in a biological framework.

## CONFLICT OF INTEREST STATEMENT

M.J.P. is currently a full‐time employee at Amprion Inc. J.R.W., A.R., H.V., I.S., D.C., C.A., M.S.B., E.N.W., H.S.O., C.B.Y., A.T., M.Y., S.J.S., V.R., R.T., K.Y., T.W.C., M.D.G., V.W.H., A.D.W., K.L.P., and E.C.M. have nothing to disclose. Author disclosures are available in the supporting information.

## CONSENT STATEMENT

All human subjects provided informed consent.

## Supporting information



Supporting Information

Supporting Information: alz71455‐sup‐0002‐SuppMat
