# Supplementary material for: Effects of α‐synuclein pathology on synaptic dysfunction and clinical outcomes in normal aging
Source: Alzheimers Dement. 2026 May 3;22(5):e71455. doi: 10.1002/alz.71455 (PMC13135916; doi:10.1002/alz.71455)
Supplement: Supplementary file 2 — Supporting Information: alz71455‐sup‐0002‐SuppMat [file ALZ-22-e71455-s002.docx]

**Supplementary Methods**

**CSF ⍺-synuclein SAA batch overview**

Overall, 472 CSF samples from 426 unique participants were sent to Amprion, Inc. After removing longitudinal data and applying exclusion criteria based on clinical characteristics, CSF a-syn SAA results from 415 total participants were included in the present cross-sectional analyses.

Batch 1: 154 samples from 153 unique participants were processed by Amprion, Inc in June 2022. 151 of these participants were included in a previous publication^1^. In this initial analysis, we identified 17 participants with unexpected clinical-biomarker profiles^1^ and re-ran the SAA (Batch 2) on a separate CSF aliquot collected during the same lumbar puncture. Three additional participant CSF samples were also processed in both batches due to study co-enrollment, for a total of 20 samples repeated across batches. Of these 20 cases assayed in both Batch 1 and Batch 2, 5 switched CSF α-syn status. We suspect status change was driven by the fact that these re-assayed CSF samples were intentional selected given their clinicobiomarker discrepancy and does not reflect the overall test-retest reliability of the a-syn SAA. Because repeat testing was not conducted across the entire cohort, ⍺-syn status for these 5 participants was defined as their Batch 1 result for analyses in the current manuscript. One MCI-AD participant processed in Batch 1 had a longitudinal CSF sample also assayed in Batch 1 (delay between CSF samples = 1.0 years); both results were CSF ⍺-syn-.

Batch 2: 318 CSF samples from 296 unique participants were processed by Amprion, Inc in January 2024. Two PD participants with baseline CSF samples in Batch 1 had longitudinal CSF samples in Batch 2 due to co-enrollment between PUC and ADRC (mean delay between samples=3.8 ± 0.3 years). These participants were CSF ⍺-syn+ at both time points. One CU participant CSF sample was included twice in Batch 2 due to co-enrollment in ADRC and SAMS; both results were CSF ⍺-syn-. One MCI-AD participant had a baseline and longitudinal CSF sample assayed in Batch 2 (delay between samples = 2.9 years); both results were CSF ⍺-syn-. Seven CU participants from SAMS had 2 longitudinal CSF samples (mean delay between samples = 5.3 ± 2.1 years) and six had 3 longitudinal CSF samples (mean delay between first and last samples = 7.3 ± 0.2 years). One CU SAMS participant had a baseline CSF sample processed in batch 1 and two longitudinal CSF samples processed in batch 2 (delay between first and last samples = 4.3 years). Of these 15 CU participants with longitudinal assessments, 13 were CSF α-syn- across all time points, and 1 was CSF α-syn- during their first visit and CSF α-syn+ 3.4 years later; for this participant all demographic and clinical data were obtained from the SAMS time point corresponding to the CSF α-syn+ result (Wave 1.5), and the individual was considered CSF α-syn+ in all analyses. One participant with a longitudinal CSF sample was deemed inconclusive at their first visit and was CSF α-syn- 7.6 years later; this participant was classified as CSF α-syn- in our analyses. A participant with a known presenilin 1 mutation was excluded. 10 participants with clinically determined AD diagnoses (MCI-AD or Dementia-AD) were excluded from analyses because they did not have evidence of CSF amyloid positivity (3 CSF Aβ-, 7 missing CSF Aβ42:Aβ40). After removing these 10 AD participants, 1 mutation carrier, the 20 CSF samples repeated across batches 1 and 2, 1 CSF sample processed twice, and 24 longitudinal CSF samples, there were a total of 262 participants processed in Batch 2 included in the manuscript.

**Supplementary Figures**

**Supplementary Figure 1. Frequencies of α-synuclein, β-amyloid, and tau positivity across clinically unimpaired participants and individuals diagnosed with Alzheimer’s disease and Lewy body disease.** Bar plot shows percentage for α-synuclein (a-syn), β-amyloid (A), and tau (T) status within each clinical group, by etiology and impairment status. A-T+ individuals (N=9) are not represented.

**Supplementary Tables**

**Supplementary Table 1 – Data availability by modality for clinically unimpaired participants.**

| **Data modality or outcome** | **Total N** | **α-syn- N** | **α-syn+ N** |
| --- | --- | --- | --- |
| **CSF α-syn SAA (full study sample)** | 269 | 245 | 24 |
| **APOE genotype** | 217 | 198 | 19 |
| **CSF β-amyloid42:β-amyloid40 ratio** | 251 | 228 | 23 |
| **CSF p-tau181** | 251 | 228 | 23 |
| **CSF YWHAG:NPTX2** | 210 | 189 | 21 |
| **Plasma GFAP & NfL** | 183 | 168 | 15 |
| **Neuropsychological testing** |  |  |  |
| Digit span forward & backward | 240 | 219 | 21 |
| Trail Making Test A | 262 | 238 | 24 |
| Trail Making Test B-A | 261 | 237 | 24 |
| HVLT-R Delayed Recall | 240 | 219 | 21 |
| Semantic Fluency | 264 | 240 | 24 |
| **MDS-UPDRS** |  |  |  |
| Part 1 (Non-motor) | 82 | 75 | 7 |
| Part 2 (Self-reported motor) | 81 | 75 | 6 |
| Part 3 (Motor exam) | 95 | 87 | 8 |
| **Neuropsychiatric Inventory Questionnaire** | 194 | 175 | 19 |

α-syn+/- refers to α-synuclein CSF seed amplification assay result. HVLT-R, Hopkins Visual Learning Test Revised; MDS-UPDRS, Movement Disorder Society-sponsored revision of the Unified Parkinson’s Disease Rating Scale.

**Supplementary Table 2 – Participants with consensus clinical diagnoses other than clinically unimpaired, Alzheimer’s disease, or Lewy body disease.**

| **Clinical diagnosis** | **Biomarker status** |
| --- | --- |
| Frontotemporal dementia | ⍺-syn-, A+T- |
| Mild cognitive impairment due to depression | ⍺-syn-, A+T- |
| Mild cognitive impairment due to cardiovascular disease | ⍺-syn-, A-T- |
| Mild cognitive impairment, not otherwise specified | ⍺-syn-, A+T+ |
| Mild cognitive impairment, not otherwise specified | ⍺-syn+, A-T- |

All five participants were enrolled in the Stanford Alzheimer’s Disease Research Center.

**Supplementary Table 3 – Alzheimer’s disease participant demographics.**

| **Cognitive status** | **Mild cognitive impairment** | | **Dementia** | |
| --- | --- | --- | --- | --- |
| **α-synuclein SAA status** | **Negative** | **Positive** | **Negative** | **Positive** |
| **N (%)** | 25 (86.2%) | 4 (13.7%) | 22 (81.4%) | 5 (18.5%) |
| **Age (years)** | 70.0 (7.86) | 74.0 (6.93) | 64.5 (8.69) | 65.0 (7.91) |
| **N (%) Female** | 7 (28.0%) | 0 (0%) | 17 (77.3%) | 3 (60.0%) |
| **Years of education** | 16.8 (2.53) | 18.0 (3.46) | 15.2 (1.92) | 17.0 (2.83) |
| Missing | 0 (0%) | 1 (25.0%) | 1 (4.5%) | 0 (0%) |
| **N (%) AD status** |  |  |  |  |
| A-T- | 0 (0%) | 0 (0%) | 0 (0%) | 0 (0%) |
| A+T- | 8 (32.0%) | 1 (25.0%) | 2 (9.1%) | 0 (0%) |
| A-T+ | 0 (0%) | 0 (0%) | 0 (0%) | 0 (0%) |
| A+T+ | 17 (68.0%) | 3 (75.0%) | 20 (90.9%) | 5 (100%) |
| **N (%) APOE genotype** |  |  |  |  |
| ε2/ε3 | 2 (8.0%) | 0 (0%) | 0 (0%) | 0 (0%) |
| ε2/ε4 | 0 (0%) | 0 (0%) | 1 (4.5%) | 0 (0%) |
| ε3/ε3 | 5 (20.0%) | 1 (25.0%) | 2 (9.1%) | 1 (20.0%) |
| ε3/ε4 | 14 (56.0%) | 0 (0%) | 10 (45.5%) | 1 (20.0%) |
| ε4/ε4 | 1 (4.0%) | 1 (25.0%) | 2 (9.1%) | 3 (60.0%) |
| Missing | 3 (12.0%) | 2 (50.0%) | 7 (31.8%) | 0 (0%) |

Participant characteristics based on clinical group and α-synuclein status. Mean and standard deviation shown for age and years of education. Alzheimer’s disease status assessed with CSF β-amyloid 42:β-amyloid 40 (cutoff <0.0943) and p-tau181 (cutoff >57.5 pg/mL). MCI, mild cognitive impairment; AD, Alzheimer’s disease; A/T, β-amyloid/tau; SAA, seed amplification assay.

**Supplementary Table 4 – Lewy body disease participant demographics.**

|  | **Cognitively unimpaired** | | **Mild cognitive impairment** | | **Dementia** | |
| --- | --- | --- | --- | --- | --- | --- |
| **CSF** **⍺-synuclein status** | **Negative** | **Positive** | **Negative** | **Positive** | **Negative** | **Positive** |
| **N (%)** | 6 (18.8%) | 32 (81.2%) | 7 (23.3%) | 23 (76.7%) | 3 (17.6%) | 14 (82.3%) |
| **Age (years)** | 68.3 (6.02) | 66.2 (7.49) | 72.4 (9.69) | 69.6 (5.79) | 74.3 (14.0) | 71.1 (6.78) |
| **N (%) Female** | 2 (33.3%) | 17 (53.1%) | 3 (42.9%) | 7 (30.4%) | 2 (66.7%) | 3 (21.4%) |
| **Years of education** | 16.8 (2.23) | 16.6 (2.27) | 18.7 (1.25) | 16.3 (2.72) | 15.3 (3.06) | 16.5 (2.18) |
| **N (%) Clinical diagnosis** |  |  |  |  |  |  |
| Parkinson’s disease | 6 (100%) | 32 (100%) | 5 (71.4%) | 20 (87.0%) | 1 (33.3%) | 10 (71.4%) |
| Dementia with Lewy bodies | 0 (0%) | 0 (0%) | 2 (28.6%) | 3 (13.0%) | 2 (33.3%) | 4 (28.5%) |
| **N (%) AD status** |  |  |  |  |  |  |
| A-T- | 5 (83.3%) | 25 (78.1%) | 4 (57.1%) | 9 (39.1%) | 0 (0%) | 3 (21.4%) |
| A+T- | 1 (16.7%) | 5 (15.6%) | 0 (0%) | 8 (34.8%) | 0 (0%) | 4 (28.6%) |
| A-T+ | 0 (0%) | 0 (0%) | 1 (14.3%) | 0 (0%) | 0 (0%) | 0 (0%) |
| A+T+ | 0 (0%) | 1 (3.1%) | 2 (28.6%) | 5 (21.7%) | 3 (100%) | 6 (42.9%) |
| Missing | 0 (0%) | 1 (3.1%) | 0 (0%) | 1 (4.3%) | 0 (0%) | 1 (7.1%) |
| **N (%) APOE genotype** |  |  |  |  |  |  |
| ε2/ε3 | 1 (16.7%) | 1 (3.1%) | 2 (28.6%) | 3 (13.0%) | 1 (33.3%) | 1 (7.1%) |
| ε2/ε4 | 0 (0%) | 1 (3.1%) | 0 (0%) | 0 (0%) | 0 (0%) | 0 (0%) |
| ε3/ε3 | 3 (50.0%) | 17 (53.1%) | 5 (71.4%) | 14 (60.9%) | 1 (33.3%) | 5 (35.7%) |
| ε3/ε4 | 1 (16.7%) | 9 (28.1%) | 0 (0%) | 5 (21.7%) | 0 (0%) | 5 (35.7%) |
| ε4/ε4 | 1 (16.7%) | 0 (0%) | 0 (0%) | 1 (4.3%) | 0 (0%) | 1 (7.1%) |
| Missing | 0 (0%) | 4 (12.5%) | 0 (0%) | 0 (0%) | 1 (33.3%) | 2 (14.3%) |

Characteristics for participants with Lewy body disease diagnoses based on cognitive impairment severity and CSF ⍺-synuclein status. Alzheimer’s disease status assessed with CSF β-amyloid 42:β-amyloid 40 and p-tau181. MCI, mild cognitive impairment; AD, Alzheimer’s disease; A/T, amyloid/tau.

**Supplementary Table 5 – Neuropsychological test performance and associations with a-synuclein SAA and p-tau181 in clinically unimpaired participants.**

| **Neuropsychological Test** | **Mean (SD)** | | **⍺-synuclein SAA**  **β ± SE, p-value** | **P-tau181**  **β ± SE, p-value** |
| --- | --- | --- | --- | --- |
|  | **⍺-synuclein SAA negative** | **⍺-synuclein SAA positive** |  |  |
| **Digit Span Forward**^2,3^ | 10.0 (2.34) | 9.71 (2.59) | 0.08 ± 0.54, 0.88 | 0.00 ± 0.01, ± 0.83 |
| **Digit Span Backward**^2,3^ | 7.75 (2.24) | 6.52 (2.23) | -0.96 ± 0.52, 0.07 | -0.01 ± 0.01, 0.18 |
| **Trail Making Test A (s)**^4^ | 30.8 (9.28) | 30.6 (5.17) | -1.07 ± 1.97, 0.59 | 0.05 ± 0.02, 0.07 |
| **Trail Making Test B-A (s)**^4^ | 41.5 (24.2) | 56.0 (34.9) | 11.2 ± 4.96, 0.02 | 0.03 ± 0.06, 0.61 |
| **HVLT-R Delayed Recall**^5^ | 9.87 (2.33) | 8.81 (3.22) | -0.69 ± 0.54, 0.20 | -0.02 ± 0.01, 0.001 |
| **Semantic Fluency**^6^ | 23.9 (6.05) | 23.3 (5.89) | -0.10 ± 1.35, 0.94 | -0.02 ± 0.02, 0.35 |

Mean (SD) values for each group are not adjusted. Linear regression models included age, sex, and years of education as covariates. Unstandardized estimates and standard error (SE) are shown.

**Supplementary Table 6 – MDS-UPDRS Part I (non-motor aspects of daily living) individual item associations with a-synuclein SAA in clinically unimpaired participants.**

| **MDS-UPDRS Part I Item** | **Mean (SD)** | | **⍺-synuclein SAA**  **β ± SE, p-value** |
| --- | --- | --- | --- |
|  | **⍺-synuclein SAA negative** | **⍺-synuclein SAA positive** |  |
| **Cognitive Impairment** | 0.19 ± 0.42 | 1.00 ± 1.15 | 0.80 ± 0.21, 0.0002 |
| **Hallucinations and Psychosis** | 0.01 ± 0.12 | 0.29 ± 0.76 | 0.28 ± 0.09, 0.004 |
| **Depressed Mood** | 0.24 ± 0.61 | 0.13 ± 0.38 | -0.10 ± 0.24, 0.69 |
| **Anxious Mood** | 0.31 ± 0.61 | 0.71 ± 0.76 | 0.46 ± 0.24, 0.06 |
| **Apathy** | 0.12 ± 0.49 | 0.57 ± 0.79 | 0.45 ± 0.21, 0.03 |
| **Features of Dopamine Dysregulation Syndrome** | 0.16 ± 0.59 | 0.00 ± 0.00 | -0.16 ± 0.22, 0.48 |
| **Sleep Problems** | 0.77 ± 1.10 | 0.57 ± 0.53 | -0.23 ± 0.43, 0.60 |
| **Daytime Sleepiness** | 0.73 ± 0.86 | 0.86 ± 0.90 | 0.02 ± 0.32, 0.94 |
| **Pain and Other Sensations** | 0.55 ± 0.87 | 1.29 ± 1.60 | 0.72 ± 0.38, 0.06 |
| **Urinary Problems** | 0.35 ± 0.83 | 0.86 ± 1.07 | 0.47 ± 0.33, 0.16 |
| **Constipation Problems** | 0.21 ± 0.60 | 0.86 ± 1.07 | 0.64 ± 0.26, 0.02 |
| **Light Headedness on Standing** | 0.13 ± 0.38 | 0.29 ± 0.49 | 0.14 ± 0.15, 0.38 |
| **Fatigue** | 0.28 ± 0.56 | 0.57 ± 0.79 | 0.30 ± 0.23, 0.34 |

All items are scored 0-4, with higher values indicating greater severity. Mean (SD) values for each group are not adjusted. Linear regression models included age and sex, as covariates. Unstandardized estimates and standard error (SE) are shown.

**Supplementary References**

1. Plastini MJ, Abdelnour C, Young CB, et al. Multiple biomarkers improve diagnostic accuracy across Lewy body and Alzheimer’s disease spectra. *Ann Clin Transl Neurol*. 2024;11(5):1197-1210. doi:10.1002/acn3.52034

2. Weintraub S, Salmon D, Mercaldo N, et al. The Alzheimer’s Disease Centers’ Uniform Data Set (UDS): The Neuropsychological Test Battery. *Alzheimer Dis Assoc Disord*. 2009;23(2):91-101. doi:10.1097/WAD.0b013e318191c7dd

3. Wechsler D. *WMS-III: Wechsler Memory Scale Administration and Scoring Manual*. Psychological Corporation; 1997.

4. Reitan RM, Wolfson D. *The Halstead-Reitan Neuropsychological Test Battery: Theory and Clinical Interpretation*. Vol 4. Reitan Neuropsychology; 1985.

5. Benedict RHB, Schretlen D, Groninger L, Brandt J. Hopkins Verbal Learning Test – Revised: Normative Data and Analysis of Inter-Form and Test-Retest Reliability. *Clin Neuropsychol*. 1998;12(1):43-55. doi:10.1076/clin.12.1.43.1726

6. Morris JC, Heyman A, Mohs RC, et al. The Consortium to Establish a Registry for Alzheimer’s Disease (CERAD). Part I. Clinical and neuropsychological assessment of Alzheimer’s disease. *Neurology*. 1989;39(9):1159-1165. doi:10.1212/wnl.39.9.1159

7. Palmqvist S, Rossi M, Hall S, et al. Cognitive effects of Lewy body pathology in clinically unimpaired individuals. *Nat Med*. 2023;29(8):1971-1978. doi:10.1038/s41591-023-02450-0

8. Tosun D, Hausle Z, Iwaki H, et al. A cross-sectional study of α-synuclein seed amplification assay in Alzheimer’s disease neuroimaging initiative: Prevalence and associations with Alzheimer’s disease biomarkers and cognitive function. *Alzheimers Dement J Alzheimers Assoc*. 2024;20(8):5114-5131. doi:10.1002/alz.13858

9. Jonaitis EM, MacLeod K, Lamoureux J, et al. Misfolded α-synuclein co-occurrence with Alzheimer’s disease proteinopathy. *Alzheimers Dement J Alzheimers Assoc*. 2025;21(5):e70205. doi:10.1002/alz.70205
